# Interplay of Environmental Factors, Genetic Susceptibility, and Sleep Disturbances predict Bipolar Disorder’s Relapses: preliminary results from a pilot study

**DOI:** 10.1192/j.eurpsy.2024.533

**Published:** 2024-08-27

**Authors:** M. Bort, G. Fico, V. Oliva, M. de Prisco, L. Bracco, C. Possidente, M. Y. Rivas, V. Ruiz, L. Montejo, E. Vieta, A. Murru

**Affiliations:** ^1^Bipolar and Depressive Disorders Unit, Hospital Clínic de Barcelona; ^2^Departament de Medicina, Facultat de Medicina i Ciències de la Salut, Universitat de Barcelona (UB); ^3^Institut d’Investigacions Biomèdiques August Pi i Sunyer (IDIBAPS), Barcelona, Spain; ^4^Department of Biomedical and Neuromotor Sciences, University of Bologna, Bologna, Italy; ^5^Centro de Investigación Biomédica en Red de Salud Mental (CIBERSAM), Instituto de Salud Carlos III, Madrid, Spain; ^6^Department of Pathophysiology and Transplantation, University of Milan, Milan, Italy; ^7^Institut Clínic de Neurociències, Barcelona, Spain

## Abstract

**Introduction:**

Predicting acute affective episodes in individuals with Bipolar Disorder (BD) remains a clinical challenge. Specific environmental stressors, including air pollution, noise, and temperature variations might worsen affective symptoms or sleep in the general population, but their role in BD relapses is often overlooked. Indeed, they might exacerbate BD by perturbing circadian rhythms – fundamental aspects of BD.

**Objectives:**

We thereby present the protocol of this pilot study and future preliminary data. We aim to longitudinally assess sleep alterations, mood fluctuations, and environmental exposure to several factors (air pollutants, climate, noise, artificial light-at-night, green space access) in patients with BD and to check the association of these variables with BD relapses.

**Methods:**

In this pilot study, we will recruit 40 patients with BD in a 6-month prospective study. Patients were assessed during baseline, at 3 and 6 months. Data recollected will consist of a subjective (questionnaires) and objective (through meteorological stations) evaluation of physical environmental factors around the home residence; clinical assessment of mood and circadian rhythms, and continuous tracking of sleep-wake patterns, energy, and movement using actigraphy.

**Results:**

Expected results will show that exposure to a worse environment (higher pollution, noise, light exposure, climate) will be associated with worse BD outcomes (i.e., relapse, mood symptoms, sleep alterations).

**Conclusions:**

We will be sharing preliminary data from our ongoing study, offering insights into early patterns and findings that shed light on our objectives.

**Disclosure of Interest:**

None Declared

